# MRI Tracking of Marine Proliferating Cells In Vivo Using Anti-Oct4 Antibody-Conjugated Iron Nanoparticles for Precision in Regenerative Medicine

**DOI:** 10.3390/bios13020268

**Published:** 2023-02-13

**Authors:** Neda Baghban, Arezoo Khoradmehr, Alireza Afshar, Nazanin Jafari, Tuba Zendehboudi, Poorya Rasekh, Leila Gholamian Abolfathi, Alireza Barmak, Gholamhossein Mohebbi, Baspakova Akmaral, Kaliyev Asset Askerovich, Mussin Nadiar Maratovich, Hossein Azari, Majid Assadi, Iraj Nabipour, Amin Tamadon

**Affiliations:** 1 The Persian Gulf Marine Biotechnology Research Center, The Persian Gulf Biomedical Sciences Research Institute, Bushehr University of Medical Sciences, Bushehr 7514633196, Iran; 2 PerciaVista R&D Co., Shiraz 7167683745, Iran; 3 MRI Department, Heart Hospital of Bushehr, Bushehr University of Medical Sciences, Bushehr 7514633196, Iran; 4 Food Laboratory, Bushehr University of Medical Sciences, Bushehr 7518759577, Iran; 5 Department for Scientific Work, West Kazakhstan Marat Ospanov Medical Unversity, Aktobe 030012, Kazakhstan; 6 General Surgery, West-Kazakhstan Medical University Named after Marat Ospanov, Aktobe 030012, Kazakhstan; 7 Nuclear Medicine and Molecular Imaging Research Center, School of Medicine, Bushehr University of Medical Sciences, Bushehr 7514633196, Iran

**Keywords:** cell proliferation, marine, MRI, tracking, magnetic nanoparticle

## Abstract

Marine invertebrates are multicellular organisms consisting of a wide range of marine environmental species. Unlike vertebrates, including humans, one of the challenges in identifying and tracking invertebrate stem cells is the lack of a specific marker. Labeling stem cells with magnetic particles provides a non-invasive, in vivo tracking method using MRI. This study suggests antibody-conjugated iron nanoparticles (NPs), which are detectable with MRI for in vivo tracking, to detect stem cell proliferation using the Oct4 receptor as a marker of stem cells. In the first phase, iron NPs were fabricated, and their successful synthesis was confirmed using FTIR spectroscopy. Next, the Alexa Fluor anti-Oct4 antibody was conjugated with as-synthesized NPs. Their affinity to the cell surface marker in fresh and saltwater conditions was confirmed using two types of cells, murine mesenchymal stromal/stem cell culture and sea anemone stem cells. For this purpose, 106 cells of each type were exposed to NP-conjugated antibodies and their affinity to antibodies was confirmed by an epi-fluorescent microscope. The presence of iron-NPs imaged with the light microscope was confirmed by iron staining using Prussian blue stain. Next, anti-Oct4 antibodies conjugated with iron NPs were injected into a brittle star, and proliferating cells were tracked by MRI. To sum up, anti-Oct4 antibodies conjugated with iron NPs not only have the potential for identifying proliferating stem cells in different cell culture conditions of sea anemone and mouse cell cultures but also has the potential to be used for in vivo MRI tracking of marine proliferating cells.

## 1. Introduction

Marine invertebrates comprise a wide range of marine species with diverse phylogenetic branches. They have a variety of organisms with simple structures, such as sponges and cnidarians to very complex ones such as mollusks, crustaceans, echinoderms, and protochordates. These organisms constitute the widest diversity on earth, with more than 2,000,000 species (95% of the total biodiversity of animals). They have been used as laboratory animals for more than 150 years and have helped to further explain various biological complexities. The following are among the important research findings on these creatures: phagocytosis in starfish larvae [1]; biological chimeras in corals [2]; the importance of sea urchins for the molecular understanding of the basis of evolutionary biology, including gene monitoring networks and controlling molecular proliferation, which led to the discovery of cyclin [3,4,5,6]; the primary safety in organisms with the colony (sponges, hydrozoans, corals, urochordates, and bryozoans); flexibility in the development and use of aquatic worms for regenerative research [7]; and the discovery of a green fluorescent protein in cnidarians [8].

Aquatic invertebrates have several stem-cell types. These stem cells allow marine invertebrates to produce large numbers of bioactive molecules. Marine stem cells play key roles in the biology of aquatic invertebrates. They are involved in aging and regeneration processes, including whole-body regeneration. Studies have shown marine invertebrate stem cells are mostly multipotent and pluripotent, but vertebrate stem cells are mostly oligopotent and unipotent [8]. In addition, unlike vertebrates, in many marine invertebrates, stem cells proliferate within the animal, meaning that they do not have a niche for their stem cells [9,10]. It is also noteworthy that transdifferentiation, which has recently attracted attention for shedding light on how to reprogram cells, exists in both groups of invertebrates with simple anatomy and complex morphology [11,12]. These observations show common and unique features of marine stem cells that may have evolved to fit the diversity of life cycle conditions, the environment, and the characteristics of marine invertebrate developmental states [8].

Nowadays, researchers have used new methods to track and follow the function of stem cells in living organisms to study the biological properties of stem cells after transplantation in living organisms. Some markers used to identify stem cells include CD271 [13], AC133 [14], Stro-1 [15], CD146+ [16], etc. Some of the common nanoparticles (NPs) used as ideal probes for non-invasive tracking of stem cells are quantum dots (QD) [17,18], silica NPs [19], and polymer NPs [20], which are mostly used for detection via fluorescence imaging; superparamagnetic iron oxide NPs (SPIONs) [21], which are used for tracking cells with magnetic resonance imaging (MRI). Au-NPs are used for photoacoustic imaging [22]. QDs, Au-NPs, or a combination of these and radioactive markers have been used to track cells using tomography methods, such as positron emission tomography (PET), single-photon emission computerized tomography (SPECT), and computerized tomography (CT) [23,24,25]. However, while there is a great deal of information about adult stem cells of vertebrates (mainly mammals) and about some of the characteristics of invertebrate models such as Drosophila sp., limited information is available about the nature and characteristics of marine stem cells [8,9], and there is a need for study in this field.

As mentioned above, marine invertebrates have a high potential for use in medicine and various biological sciences due to their valuable metabolites. Although studies on marine stem cells have increased dramatically in recent years, the detection and identification of marine invertebrate stem cells have not been investigated effectively. Due to the importance of identifying and tracking marine invertebrate stem cells and the lack of study in this field, this study was proposed to suggest a non-invasive method to detect marine cell proliferation in vitro using anti-Oct4 antibodies conjugated with iron NPs, which can also be used for identifying and tracking marine invertebrate stem cells in vivo via MRI. To the best of our knowledge, there has not yet been any report on the detection of marine cell proliferation using a specific marker, and all reports have been in the field of vertebrates. It is also the first report on the synthesis of the Alexa Fluor^®^ 594 antibody conjugated with iron NPs, which are detectable using both MRI and fluorescence methods. Moreover, this is the first study on conjugating Oct4 antibodies with iron NPs and on the possibility of tracking it in brittle stars.

## 2. Results

### 2.1. Synthesis of Fe_3_O_4_@SiO_2_

**Figure 1:**
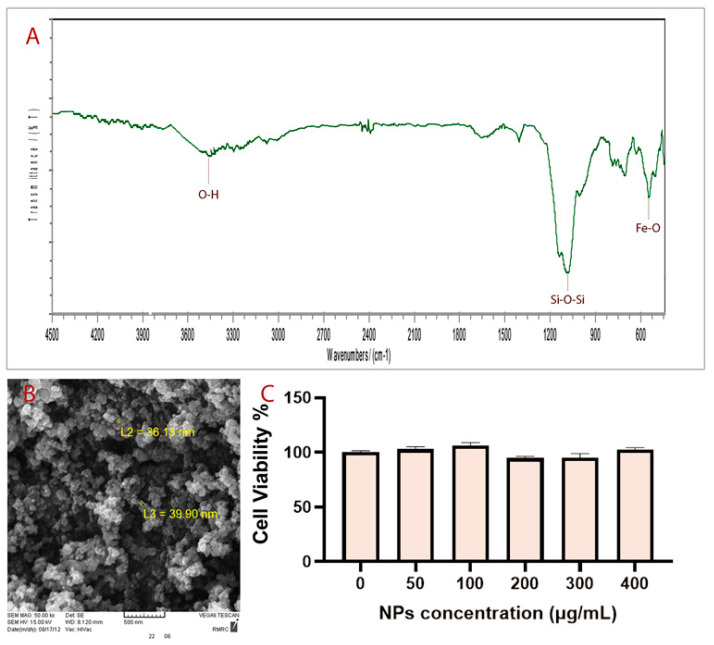
(**A**) FT-IR spectrum, (**B**) SEM image, and (**C**) MTT analysis.

The fabrication of Fe_3_O_4_@SiO_2_ NPs was performed through a continuous two-step process. In the first step, Fe_3_O_4_ NPs were fabricated by coprecipitation of Fe3+ and Fe^2+^ with a mole ratio of 2:1 [26]. In the second step, Fe_3_O_4_ NPs were coated with a silica layer to stabilize them and to prevent them from agglomerating [27]. FT–IR spectrum corresponding to the synthesized NPs is shown in Figure 1A, which shows several specific bands at approximately 3500 cm^−1^, 1100 cm^−1^ and 575 cm^−1^. The average size of particles obtained is about 38 nm according to SEM analysis (Figure 1B). The in vitro cell toxicity assay (Figure 1C) and in vivo toxicity assay of synthesized Fe_3_O_4_@SiO_2_ NPs showed that NPs are not toxic at the studied dosages. Moreover, the microscopy analysis of as-synthesized NPs using different fluorescent filters showed that they have no autofluorescence properties (Figure 1).

### 2.2. Anti-Oct4 Was Conjugated with Fe_3_O_4_@SiO_2_ Nanoparticles

**Figure 2:**
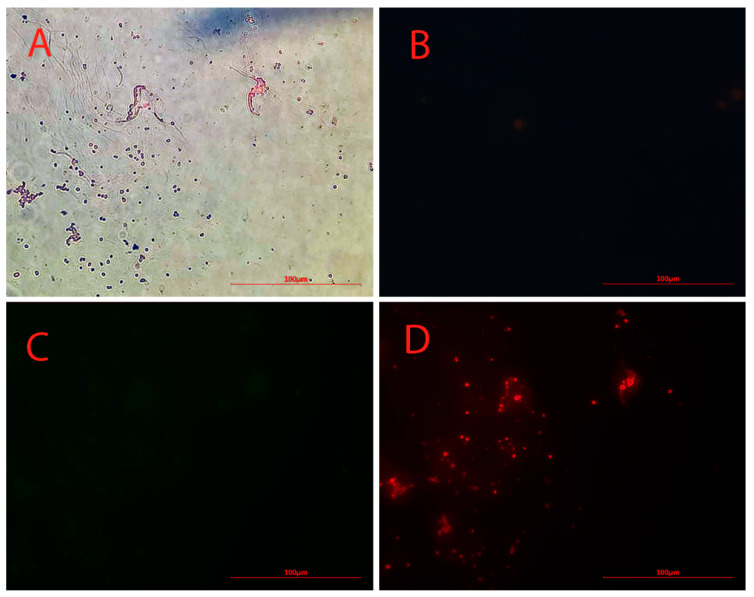
Microscopy images of Alexa Flour 594 anti-Oct4 antibody-conjugated nanoparticles using (**A**) brightfield, (**B**) blue filter, (**C**) green filter, and (**D**) red filter.

The dark points in Figure 2A clearly indicate the presence of iron particles. No green or blue fluorescence was observed under green and blue radiation for anti-Oct4-conjugated NPs (Figure 2B,C). However, points with red fluorescence, whose location matches the location of dark points in Figure 2D, were clearly observed by using a red filter.

### 2.3. Conjugated NP Detected Both Mouse and Sea Anemone Proliferating Cells

**Figure 3:**
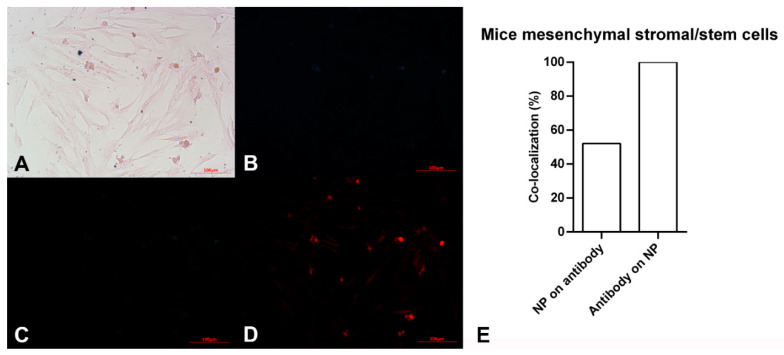
Microscopy images of mouse mesenchymal stromal/stem cells after exposing to Alexa Flour 594 anti-Oct4 antibody-conjugated nanoparticles using (**A**) brightfield, (**B**) blue filter, (**C**) green filter, and (**D**) red filter. (**E**) co-localization of conjugated iron nanoparticle staining and anti-Oct4 antibody in mouse mesenchymal stromal/stem cells. Columns show Manders’ coefficients expressed as percent values.

**Figure 4:**
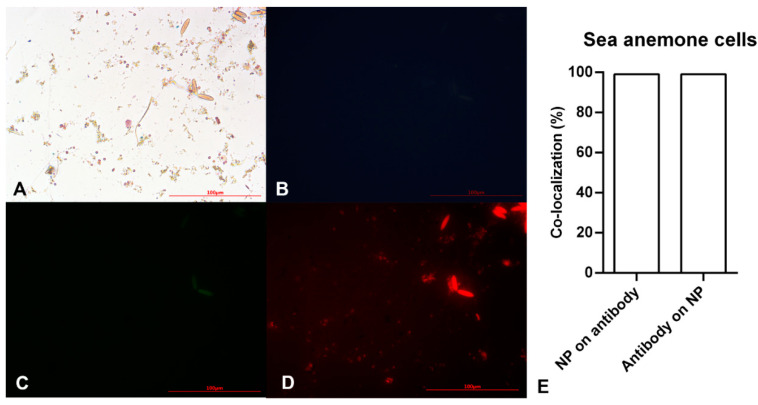
Microscopy images of sea anemone stem cells after exposing to Alexa Flour anti-Oct4 antibody-conjugated iron nanoparticles using (**A**) brightfield, (**B**) blue filter, (**C**) green filter, and (**D**) red filter. (**E**) Co-localization of conjugated iron nanoparticle staining and anti-Oct4 antibody in sea anemone stem cells. Columns show Manders’ coefficients expressed as percent values.

In order to study and confirm proliferating stem cells, two types of stem cells treated with anti-Oct4-conjugated NPs were studied using a light microscope and an epi-fluorescent microscope. The images obtained for mouse and sea anemone stem cells have been presented in Figure 3 and Figure 4, respectively.

The pink spindle-shaped plastic-adherent cells observed in Figure 3A indicate the mouse mesenchymal stromal/stem cells. The dark blue points confirm the presence of iron particles. As expected, no fluorescent light was observed using blue and green filters (Figure 3B,C). However, points with red fluorescence are observed in Figure 3D, which is related to the red filter image. This observation shows the red fluorescent emissions are not auto-fluorescent. These are assigned to Alexa Fluor 594 dyes bound to anti-Oct4. It is hypothesized that anti-Oct4 attaches to cells via Oct4, which is expressed by cells. Therefore, the presence of anti-Oct4 verifies the proliferation of cells.

After ensuring the efficiency of the as-prepared material in detecting mouse stem cell proliferation, a similar study was conducted on sea anemone stem cells to detect in vitro marine stem cell proliferation. Figure 4A demonstrates small brownish-yellow circular and oval cells. The small circular cells become pink with a dark blue layer around them after staining with the Prussian Blue stain. Pink and blue colors show cells and iron particles, respectively. These cells showed no blue or green fluorescent emission (Figure 4B,C), instead emitting red fluorescence. On the other hand, oval cells showed auto-fluorescent emission (Figure 4B,C). Therefore, the circular points with red fluorescent are Alexa Fluor^®^ 594 anti-Oct4-conjugated NPs attaching to cells via Oct4. This clearly showed sea anemone stem cell proliferation.

According to the results of image analysis of mouse MSCs using ImageJ software, 100% of anti-Oct4 overlapped with iron NPs and 52% of iron NPs overlapped with anti-Oct4. Furthermore, in the sea anemone stem cells, 99% of anti-Oct4 overlapped with iron and 99% of iron NPs overlapped with anti-Oct4 (Figure 3E and Figure 4E).

### 2.4. Conjugated NPs Were Used for In Vivo MRI Tracking of Brittle Star

**Figure 5:**
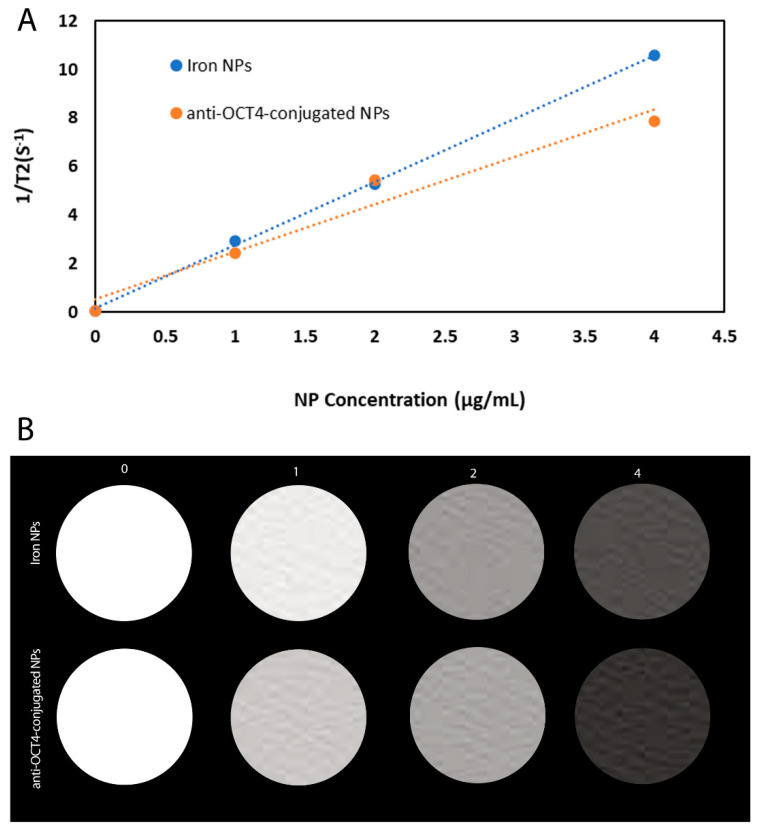
(**A**) Plot of 1/T2 versus NP concentration for iron NPs and anti-Oct4-conjugated NP, (**B**) The respective relaxivity bar diagram for iron NPs and anti-Oct4-conjugated NP.

We have carried out MR transverse relaxivity (r2) measurements of NPs and anti-Oct4-NPs for the evaluation of their MRI performance. Figure 5A and Figure 4B represent the linear plot of the transverse relaxation rate (R2) vs. NP concentration and the corresponding relaxivity bar diagram for iron NPs and anti-Oct4-conjugated NP.

**Figure 6:**
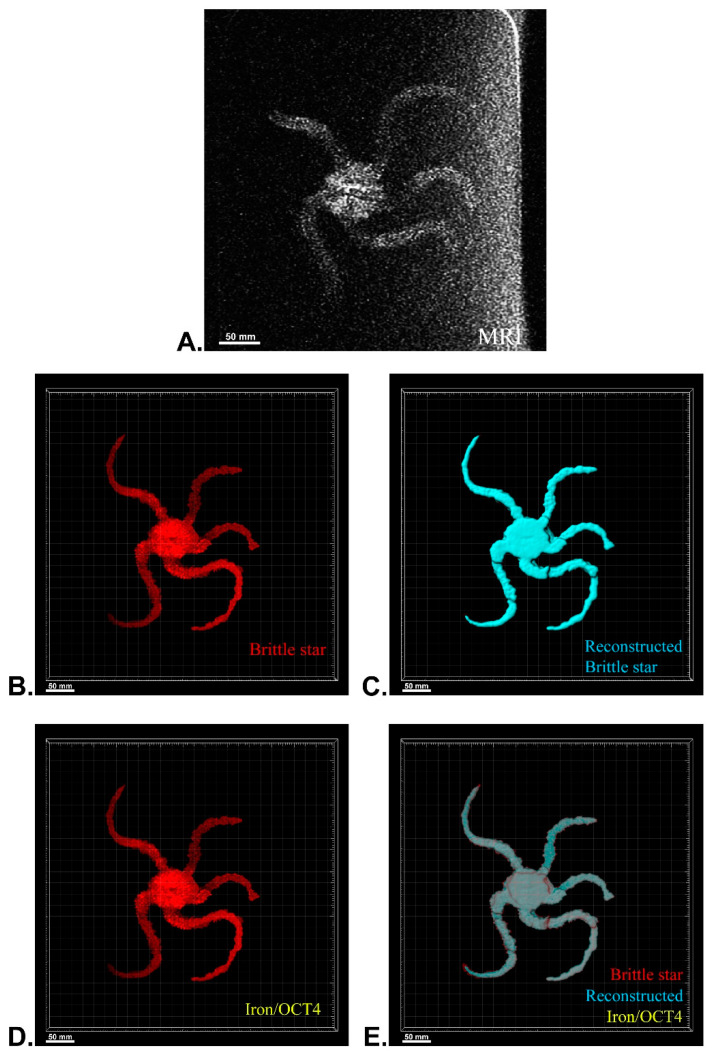
Three-dimensional (3D) reconstruction of brittle star tissue with no treatment using magnetic resonance imaging (MRI) serial images: (**A**) original grayscale image; (**B**) whole tissue imaging and signals of the brittle star tissue without reconstruction; (**C**) brittle star whole tissue 3D reconstruction in which the blue surface represents reconstructed brittle star tissue; (**D**) 3D reconstruction of the iron/iron plus anti-Oct4 signals, which is negative in this image; (**E**) 3D reconstruction of the whole tissue of brittle star along with all signals. All scale bars in four images are 50 mm. All images were processed and generated by Imaris software (V 7.4.2, ImarisX64; Bitplane AG, Schlieren, Switzerland).

**Figure 7:**
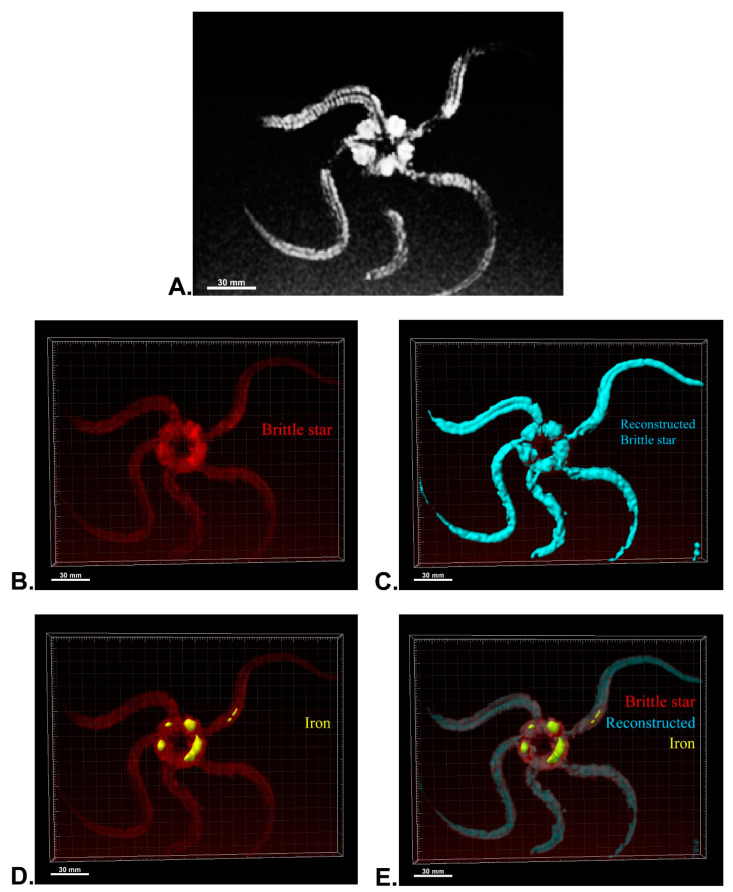
Three-dimensional (3D) reconstruction of brittle star tissue treated with iron using magnetic resonance imaging (MRI) serial images: (**A**) original grayscale image; (**B**) whole tissue imaging and signals of the brittle star tissue without reconstruction; (**C**) brittle star whole tissue 3D reconstruction in which the blue surface represents reconstructed brittle star tissue; (**D**) 3D reconstruction of the iron signals (shown in yellow); (**E**) 3D reconstruction of the whole tissue of brittle star with its all signals. All scale bars in four images are 30 mm. All images were processed and generated by Imaris software (V 7.4.2, ImarisX64; Bitplane AG).

**Figure 8:**
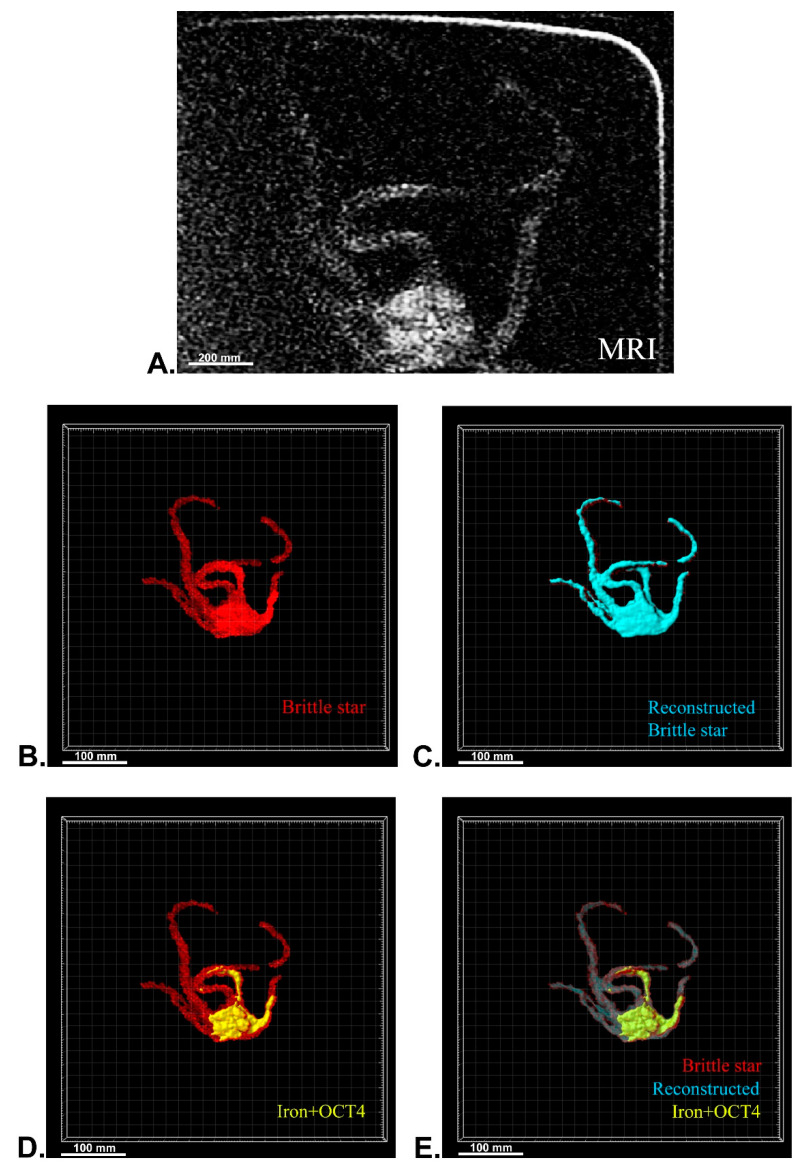
Three-dimensional (3D) reconstruction of brittle star tissue with iron plus anti-Oct4 antibody treatment using magnetic resonance imaging (MRI) serial images: (**A**) original grayscale image; (**B**) whole tissue imaging and signals of the brittle star tissue without reconstruction; (**C**) brittle star whole tissue 3D reconstruction in which the blue surface represents reconstructed brittle star tissue; (**D**) 3D reconstruction of the iron plus anti-Oct4 antibody signals (shown in yellow); (**E**) 3D reconstruction of the whole tissue of brittle star with its all signals. All scale bars in four images are 100 mm. All images were processed and generated by Imaris software (V 7.4.2, ImarisX64; Bitplane AG).

After MRI processing of three brittle stars (negative control, iron treatment, anti-Oct4-conjugated NP treatment), the brittle star tissues were successfully reconstructed by Imaris software. Four types of images were obtained for each brittle star, including whole tissue imaging and signals of the brittle star tissue without reconstruction, the brittle star whole tissue 3D reconstruction, 3D reconstruction of the iron/anti-Oct4-conjugated NP signals, and 3D reconstruction of the whole tissue of brittle star along with all signals (Figure 6, Figure 7 and Figure 8). As expected, no signal was observed in the 3D reconstruction of the whole tissue or of the whole tissue with all signals for the negative control brittle star. 3D reconstruction of the brittle star tissues after injection of iron NPs and anti-Oct4-conjugated NPs showed positive signals that appear in yellow. Positive signals were strong in the disk of both treated brittle stars as well as in the initial part of two arms of brittle stars treated with anti-Oct4-conjugated NP.

## 3. Discussion

Fe_3_O_4_@SiO_2_ NPs have been successfully synthesized. FT–IR spectra of prepared NPs show specifically that the broadband at about 3500 cm^−1^ matched with the O–H stretching vibration of silanol groups. The strongest band at about 1100 cm^−1^ corresponds to Si–O–Si bending. The peak observed at about 575 cm^−1^ is related to Fe-O bending.

The microscopy study showed anti-Oct4 was successfully conjugated with Fe_3_O_4_@SiO_2_ NPs. In previous studies, iron NPs and Alexa Fluor dyes were separately conjugated to antibodies for detecting stem cells using MRI and fluorescent techniques [28,29,30,31,32]. In the current study, we conjugated Alexa Fluor^®^ 594 anti-Oct4 with Fe_3_O_4_@SiO_2_ NPs, which not only allowed us to track stem cells using both techniques of MRI and fluorescent but also let us perform the conjugation step without using an expensive linker, because of the presence of Alexa Fluor^®^ 594 in the structure of the secondary antibody.

As Oct4 is naturally expressed in stem cells, it can be used as a marker to identify stem cells [33,34] and stem cell-like cells [35,36]. Therefore, in the current study, the presence of anti-Oct4 in microscopy images of stem cells treated with anti-Oct4-conjugated NPs verifies the proliferation of mouse and sea anemone cells. It is worth noting that the expression of Oct4 by marine cells has been reported previously [37]. Accordingly, it has been used as a marker to detect marine stem cell proliferation in the current study.

These results confirmed the functionality of conjugated iron NPs in mouse MSCs and sea anemone cells. The percentage of signal overlapping in mouse cells means that all antibodies were conjugated with iron NPs, but 48% of the iron NPs were free; this is because the amount of iron exceeded the stoichiometric amount relative to anti-Oct4. On the other hand, in the sea anemone sample, similar co-localization of NPs and antibodies for the detection of proliferating cells confirms that all antibodies were conjugated with iron NPs and there were no free iron NPs. As there are fewer sea anemone cells, almost no cells are available for attachment to the excess iron NPs and these were removed through the washing process.

Several morphological studies have been performed on marine creatures such as horseshoe crab [38,39], crab [40,41], and starfish [42,43] using MRI, but no studies have been reported for MRI tracking of marine creatures, even though many studies have been performed on the application of antibody-conjugated iron NPs for in vitro and in vivo MRI. In this study, conjugated NPs were successfully used for in vivo MRI tracking of brittle star. The high density of signals at the disk area can be attributed to the point of injection. Moreover, the high density of signals in the initial parts of two arms of the bitter star treated with anti-oct4 conjugated NP can be attributed to their adjustment to the place of injection.

## 4. Materials and Methods

### 4.1. Materials

All chemicals, buffers, and solvents were prepared with analytical grade from Merck (Merck company, Darmstadt, Germany) or Sigma (Sigma–Aldrich, St. Louis, MO, USA). Alexa Fluor^®^ 594 anti-Oct4 antibody was purchased from BioLegend (San Diego, CA, USA).

### 4.2. Preparation of Iron Nanoparticles Conjugated with Anti-Oct4 Antibody

**Figure 9:**
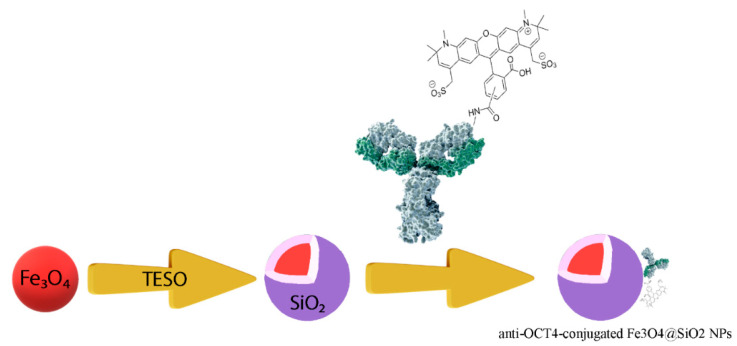
The procedure for preparing anti-Oct4 antibody-conjugated iron nanoparticles. TEOS: tetraethyl orthosilicate.

Figure 9 shows schematically the procedure for preparing anti-Oct4-conjugated iron NPs. This procedure is described in the following:

### 4.3. Synthesis of Fe_3_O_4_@SiO_2_

Fe_3_O_4_@SiO_2_ NPs were synthesized through the following steps: in the first step, Fe_3_O_4_ NPs were fabricated by dissolving 0.8100 g FeCl3.6H2O and 0.4170 g FeSO4.7H2O in 50 mL distilled water in a 200 mL round-bottom flask while it was vigorously stirred at 80 °C under argon gas. Next, 20 mL of a concentrated ammonia solution was gradually poured into the flask. In this step, Fe_3_O_4_ NPs were formed. The bottle was closed to prevent the entrance of air to the reaction container during the synthesis procedure and it was left for 1 h at 80 °C. It was then washed three times with distilled water [44].

In the next step, Fe_3_O_4_@SiO_2_ was prepared. For this purpose, 140 mL ethanol and 5 mL NH_3_ were added to the bottle containing Fe_3_O_4_ NPs and the bottle was placed on a heater stirrer at a temperature of 40 °C for 10 min under vigorous stirring. A solution containing ethanol (20 mL) and tetraethyl orthosilicate (TEOS) (3 mL) was slowly added to the reaction bottle under vigorous stirring at ambient temperature. The obtained Fe_3_O_4_@SiO_2_ NPs were then rinsed with distilled water and acetone [45,46]. The particles were stored as an ethanolic suspension.

### 4.4. Characterization of Fe_3_O_4_@SiO_2_ Nanoparticles

In order to study the successful synthesis of Fe_3_O_4_@SiO_2_ NPs, the FT–IR spectrum of prepared NPs was recorded on a Rayleigh WQF-510 FTIR spectrometer using the KBr technique. The region of 500–4500 cm^−1^ was set for measuring the spectrum. To study the size of the synthesized particles, a SEM image was prepared.

### 4.5. In Vitro Cell Toxicity of Fe_3_O_4_@SiO_2_ Nanoparticles

In order to test in vitro cell toxicity of prepared iron NPs, they were evaluated using an MTT (3-[4,5-dimethylthiazol-2-yl]-2,5-diphenyltetrazolium bromide) assay [47]. A density of 4 × 104 HFF cells per well in 200 μL DMEM culture medium were seeded into 18 wells of a 96-well plate and incubated for 24 h at 37 °C under a 5% CO_2_ atmosphere. The cells were then treated with different amounts of NPs including 0, 50, 100, 200, 300, and 400 μg/mL and incubated for 72 h. After that time, the culture medium was removed and 100 μL of the MTT solution at a concentration of 0.5 mg/mL was added to the wells and kept for a further 4 h at 37 °C. Next, MTT formation was dissolved in 100 μL of DMSO, and its absorbance was read at 570 nm using ELISA (Synergy™ 2 Multi-Mode Microplate Reader; Biotek, Beijing, China).

### 4.6. In Vivo Toxicity of Fe_3_O_4_@SiO_2_ Nanoparticles

In order to test in vivo toxicity of different dosages of prepared iron NPs, 12 bitter stars were selected and divided into 4 groups containing 3 bitter stars. Fe_3_O_4_@SiO_2_ NPs at a final dosage of 1, 3, and 5 μg in 1 mL phosphate-buffered saline (PBS) were injected in to 3 groups through disks. They were checked and compared with the control group every 24 h for 72 h. Three bitter stars were used as controls without injection.

### 4.7. Conjugation of Anti-Oct4 with Fe_3_O_4_@SiO_2_ Nanoparticles

4 c of Fe_3_O_4_@SiO_2_ NPs were mixed with 1 mL of 0.01 mol/L PBS solution (pH = 7.4), and then 0.25 μg anti-Oct4 (0.5 μL) were added to the mixture. The mixture was vortexed for 1 min and kept overnight at 4°C. The anti-Oct4 antibody-conjugated NPs were then separated using an external magnet (1.2 T, 10 cm × 5 cm × 2 cm) and washed 3 times with PBS and stored in 1.0 mL of 0.01 mol/L PBS at 4 °C until use. This was examined through a fluorescent microscope and its FT–IR spectrum was provided for further study of the bonding mechanism.

### 4.8. Cell Culture

Mouse bone marrow cells were prepared from Stem Cells Technology Research Center, Shiraz University of Medical Sciences, Shiraz, Iran. In order to culture sea anemone cells, 5–10 tentacles were isolated from an Entacmaea quadricolor, a species of sea anemone collected from the Persian Gulf, Bushehr, Iran. These tentacles were put in 0.2 µm filtered, sterile, calcium-free, artificial seawater and rinsed with 0.2 µm filtered sterile artificial seawater (SASW) enriched with a 1% penicillin–streptomycin solution (Gibco™, Scotland, UK) and amphotericin B. Tentacle cells were seeded in a T-25 tissue culture flask (NEST, China) containing 3 mL culture medium (20% Dulbecco’s Modified Eagle’s medium DMEM (Gibco™, UK), 5% fetal bovine serum (Kiazist, Iran), 2% penicillin–streptomycin solution (Gibco™, UK), 2% amphotericin B, and 71% SASW) and cultured in a CO2 incubator (Shimaz, Iran) in the dark at a temperature of 20 °C. The culture medium was replaced 3 days after culturing and the medium was then replaced weekly until it had cultured for 17 days [48,49].

### 4.9. Interaction of cells with Antibody-Conjugated Magnetic Nanoparticles

A density of 5 × 10^4^ cells of both cell types (mouse bone marrow and sea anemone cells) per well in 200 μL DMEM culture medium was seeded into 4 wells of a 96-well plate (two replicates for each cell type) and incubated 24 h at 37 °C under a 5% CO_2_ atmosphere. These cells were then fixed using 4% paraformaldehyde. Afterward, in order to study cell proliferation through detecting the expression of Oct4, the DMEM culture medium was replaced with a 200 μL solution containing 100 μL DMEM culture medium and 100 μL as-prepared anti-Oct4 antibody-conjugated NPs. After storing this mixture overnight at 4 °C, the culture medium was removed, and cells were washed using PBS three times to remove non-attached antibody-conjugated NPs. The cells were then examined with an epi-fluorescent microscope.

### 4.10. Prussian Blue Staining

In order to confirm the presence of iron NPs, the Prussian blue stain (Iron staining kit; AmirPayvand, Tehran, Iran) was used. Briefly, cells attached to anti-Oct4 antibody-conjugated NPs were fixed using a 4% formaldehyde solution. Equal parts of 20% hydrochloric acid and 10% potassium ferrocyanide solution were then mixed before use. This mixture was poured into the wells containing fixed cells and after 15 min was removed and completely washed using distilled water. Safranin O was poured into the wells and removed after 5 min. The cells were examined using a microscope after washing with distilled water. The blue, red, and pink indicate iron (ferric form), nuclei, and cytoplasm, respectively.

### 4.11. Image Analysis of Co-Localization of Conjugated Iron Nanoparticle Staining and Anti-Oct4 Antibody

Image analysis of the mouse mesenchymal stromal/stem cells and sea anemone stem cells, co-localization of conjugated iron nanoparticle staining, and anti-Oct4 antibodies was performed using ImageJ software (64-bit Java 1.8.0_172; US National Institutes of Health). In detail, two microscopy images of mouse mesenchymal stromal/stem cells with a red filter and without a filter were opened. The RGB color images were then converted to 8-bit by using “Image type” in the “Image” panel. The brightness and contrast of the image were then adjusted. After that, the Just Another Colocalization (JACoP) Plugin was selected from the “Plugins” panel to analyze the images. Furthermore, the same processes were used for analyzing the microscopy images of sea anemone stem cells with a red filter and without a filter.

### 4.12. Relaxivity

Relaxivity measurements were made on a 1.5 T clinical MRI system (SIGNA, USA) using a Flex receiver coil (GE Healthcare, Chicago, IL, USA). Acquisition parameters used for R2 measurements are as follows: TR = 2000, TE = 76, and slice thickness = 0.6.

### 4.13. MRI of Brittle Stars

**Figure 10:**
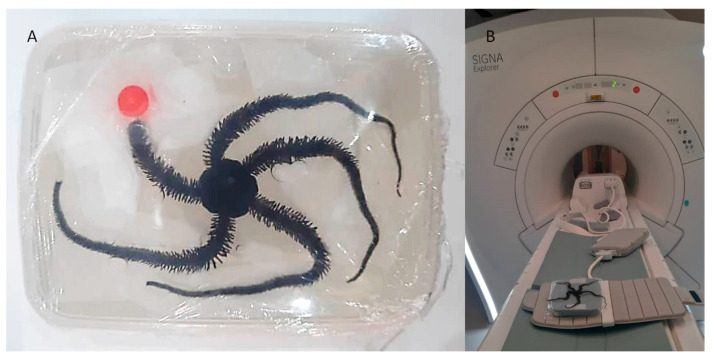
Procedure of MRI imaging of brittle star. (**A**) The brittle star was placed in a plastic container (10 cm × 15 cm × 5 cm) for MRI procedures. (**B**) The SIGNA 1.5 T MRI portfolio and Flex coil for arm imaging were used for MRI procedures.

For MRI processing, three brittle stars, Ophiocoma cynthiae, were collected from the Persian Gulf (Bushehr, Iran) and kept in a saltwater aquarium until testing. To investigate the ability of in vivo detection of iron NPs and anti-Oct4 antibody-conjugated NPs, 1 mL of these (containing 2 μg NP) were injected into the disk of brittle stars, which underwent MRI scanning after 45 min. For MRI, the brittle stars were placed into a closed plastic container filled with 30% sea water and 70% ethanol. The plastic container size of 10 cm × 15 cm × 5 cm was selected, as the receiver coil could be located as close as possible to the scanned brittle star to attain an appropriate signal-to-noise ratio. The brittle stars were scanned by a SIGNA 1.5 T clinical MRI system (United States) (Figure 10). A Flex receiver coil (GE Healthcare, Chicago, IL, USA) for arm imaging and a HypereCube T2 scanning sequence were used.

### 4.14. 3D Reconstruction

Using ImageJ software (ImageJ, U. S. National Institutes of Health, Bethesda), serial images of MRI were imported into ImageJ software and were combined as a TIFF series image using the “Images to Stack” tool in the “image” panel. The TIFF series image was saved in TIFF format. Then, 3D reconstruction of MRI images of brittle stars was performed using Imaris software (V 7.4.2, ImarisX64; Bitplane AG). Specifically, after importing the serial TIFF image, the scale of the image was corrected. In detail, the z-stack of the images was corrected by thickness for each MRI image using “Image properties” in the “Edit” panel. Firstly, the whole image was reconstructed using the “Surfaces” algorithm. In order to reconstruct other structures, the “Surfaces” algorithm was again used with different colors. The blue “Surfaces” algorithm was used to reconstruct the whole tissue of brittle stars and the yellow “Surfaces” algorithm was used for brittle stars’ tissue, which was labeled with iron or iron-conjugated Oct4 antibody.

### 4.15. Statistical Analysis

All data are reported in terms of Manders’ coefficients expressed as percent values. Charts were created by GraphPad Prism, version 5.01 for Windows (GraphPad Inc., San Diego, CA, USA).

## 5. Conclusions

In the current research, non-toxic Fe_3_O_4_@SiO_2_ NPs were fabricated through a two-step process. Alexa Flour anti-Oct4 antibody-conjugated NPs were successfully attached to mouse mesenchymal stromal/stem cells and to sea anemone stem cells, and their proliferation was confirmed. To the best of our knowledge, this is the first time that this method has been used for confirming the proliferation of marine stem cells. In addition to this exceptional achievement, attaching the iron particles to the Alexa Fluor antibody provides cost-effective antibody-conjugated NPs, which are detectable using both fluorescence and MRI methods. Therefore, they can be used for in vivo detecting and tracking stem cells in future studies.

## Data Availability

Data contained within the article and row datasets related to this project can be obtained from the corresponding author based on a reasonable request.
